# The NOD Mouse Beyond Autoimmune Diabetes

**DOI:** 10.3389/fimmu.2022.874769

**Published:** 2022-04-29

**Authors:** Anne-Marie Aubin, Félix Lombard-Vadnais, Roxanne Collin, Holly A. Aliesky, Sandra M. McLachlan, Sylvie Lesage

**Affiliations:** ^1^ Immunology-Oncology Division, Maisonneuve-Rosemont Hospital Research Center, Montreal, QC, Canada; ^2^ Département de Microbiologie, Infectiologie et Immunologie, Université de Montréal, Montréal, QC, Canada; ^3^ Department of Microbiology and Immunology, McGill University, Montreal, QC, Canada; ^4^ CellCarta, Montreal, QC, Canada; ^5^ Thyroid Autoimmune Disease Unit, Cedars-Sinai Research Institute, Los Angeles, CA, United States; ^6^ Department of Medicine, David Geffen School of Medicine at University of California Los Angeles (UCLA), Los Angeles, CA, United States

**Keywords:** NOD mice, polyautoimmunity, thyroiditis, neuropathy, biliary disease

## Abstract

Autoimmune diabetes arises spontaneously in Non-Obese Diabetic (NOD) mice, and the pathophysiology of this disease shares many similarities with human type 1 diabetes. Since its generation in 1980, the NOD mouse, derived from the Cataract Shinogi strain, has represented the gold standard of spontaneous disease models, allowing to investigate autoimmune diabetes disease progression and susceptibility traits, as well as to test a wide array of potential treatments and therapies. Beyond autoimmune diabetes, NOD mice also exhibit polyautoimmunity, presenting with a low incidence of autoimmune thyroiditis and Sjögren’s syndrome. Genetic manipulation of the NOD strain has led to the generation of new mouse models facilitating the study of these and other autoimmune pathologies. For instance, following deletion of specific genes or *via* insertion of resistance alleles at genetic loci, NOD mice can become fully resistant to autoimmune diabetes; yet the newly generated diabetes-resistant NOD strains often show a high incidence of other autoimmune diseases. This suggests that the NOD genetic background is highly autoimmune-prone and that genetic manipulations can shift the autoimmune response from the pancreas to other organs. Overall, multiple NOD variant strains have become invaluable tools for understanding the pathophysiology of and for dissecting the genetic susceptibility of organ-specific autoimmune diseases. An interesting commonality to all autoimmune diseases developing in variant strains of the NOD mice is the presence of autoantibodies. This review will present the NOD mouse as a model for studying autoimmune diseases beyond autoimmune diabetes.

## Highlights

1. The Non-Obese Diabetic (NOD) mouse as a model of multiple autoimmune diseases
2. Congenic and transgenic NOD mice represent relevant models of human pathologies
3. Spontaneous occurrence of autoimmune thyroiditis, neuropathy and biliary diseases
4. The NOD mouse can be used to study polyautoimmune phenotypes

## Introduction to the Non-Obese Diabetic Mouse Strain

Since its first description by Makino et al. in 1980 (1), the Non-Obese Diabetic (NOD) mouse strain represents the only mouse model that spontaneously develops autoimmune diabetes (2–4). The NOD strain is originally derived from Cataract Shinogi (CTS) mice, an inbred subline of the outbred ICR mouse strain, which develop cataracts (1, 2). In an effort to generate a mouse model for insulin-dependent diabetes, CTS mice with either low or high fasting glucose levels were further interbred. Eventually, mice from the ‘normoglycemic’ colony presented with diabetic symptoms, namely polyuria and glycosuria. These mice were selected for breeding, establishing the original NOD mouse colony (1, 5). Importantly, the autoimmune diabetes pathology in NOD mice shares several characteristics with human type 1 diabetes (T1D) (1, 3, 6, 7). For instance, the major histocompatibility class (MHC) locus is a defining autoimmune diabetes susceptibility factor in both mice and humans, with a common amino acid substitution in an MHC class II gene (4, 8). Studying the NOD mouse has considerably improved our understanding of this autoimmune disease, facilitating the identification of genetic variants contributing to disease susceptibility, of various immune cells causing pancreatic β-cell destruction, and of environmental contributors to disease susceptibility (6, 7, 9, 10). For further information on the use of NOD mice in dissecting the pathophysiology of autoimmune diabetes, the readers are referred to the following reviews on the topic (2–4).

This review will instead focus on the other organ-specific autoimmune diseases that spontaneously develop in NOD mice as well as in genetically manipulated NOD mice. Specifically, several NOD congenic mice and NOD genetic knockout mice are protected from autoimmune diabetes. In these diabetes-resistant mice, other autoimmune diseases spontaneously arise, such as autoimmune thyroiditis, autoimmune polyneuropathies, and autoimmune biliary disease. The use of the NOD mouse and its variants to study polyautoimmune syndromes will also be discussed. While autoantigen-specific T cell responses are a critical part of the pathology in autoimmune diabetes (6, 11), this review will more broadly discuss the presence of immune cells in the target tissues as well as the presence of autoantibodies in variants of the NOD mouse model, for each autoimmune pathology.

## Autoimmune Thyroid Disease

Autoimmune thyroid disease (AITD) includes Hashimoto’s thyroiditis, Graves’ disease (autoimmune hyperthyroidism), neonatal Graves’ disease, and postpartum thyroiditis (12). All forms of AITD are characterized by the presence of immune infiltrates (in variable amounts) in the thyroid gland and particularly by the presence of IgG class autoantibodies directed towards specific thyroid autoantigens, namely thyroglobulin, thyroid peroxidase (TPO), and the thyrotropin receptor (TSHR) (13). Of note, while some of these autoantibodies are present in the serum of many individuals with normal thyroid function, the presence of TPO is significantly associated with thyroid disease (14). Interestingly, the prevalence of AITD is more frequent in people living with T1D (PWT1D) than in the general population (15–18). Based on the study of Hwang et al., the prevalence of thyroglobulin and TPO thyroid autoantibodies in PWT1D is around 30% (19), whereas the prevalence in the general population is approximately 10% (14).

### The NOD Mouse as a Model of Autoimmune Thyroiditis

As in PWT1D, NOD mice can develop spontaneous autoimmune thyroiditis (SAT). In NOD mice, the cumulative incidence at one year ranges from ~5% to ~15% (20, 21). In both humans and mice, an iodine-rich diet accelerates the development of the disease (21, 22). The iodide excess is toxic for thyroid cells by a mechanism involving oxidative stress (23). This parallel between humans and mice highlights the relevance of the NOD mouse model for understanding autoimmune thyroiditis pathology (24). However, there are limits associated with the use of the NOD mice for the study of SAT. For one, NOD mice have a high incidence of autoimmune diabetes, especially in females where it reaches 70 to 90% by 30 weeks (25). This presents a challenge when attempting to isolate the immunological factors that specifically drive SAT independently of the immune response to autoimmune diabetes. In addition, the incidence of SAT is low in NOD mice in absence of an iodine-rich diet (20), such that very large cohorts of mice must be used to characterize the progression of the pathophysiology. Currently, an autoimmune diabetes-resistant genetic derivative of the NOD mouse model, the NOD.*H2^h4^* congenic mouse, is more commonly used to study SAT.

**Figure 1:**
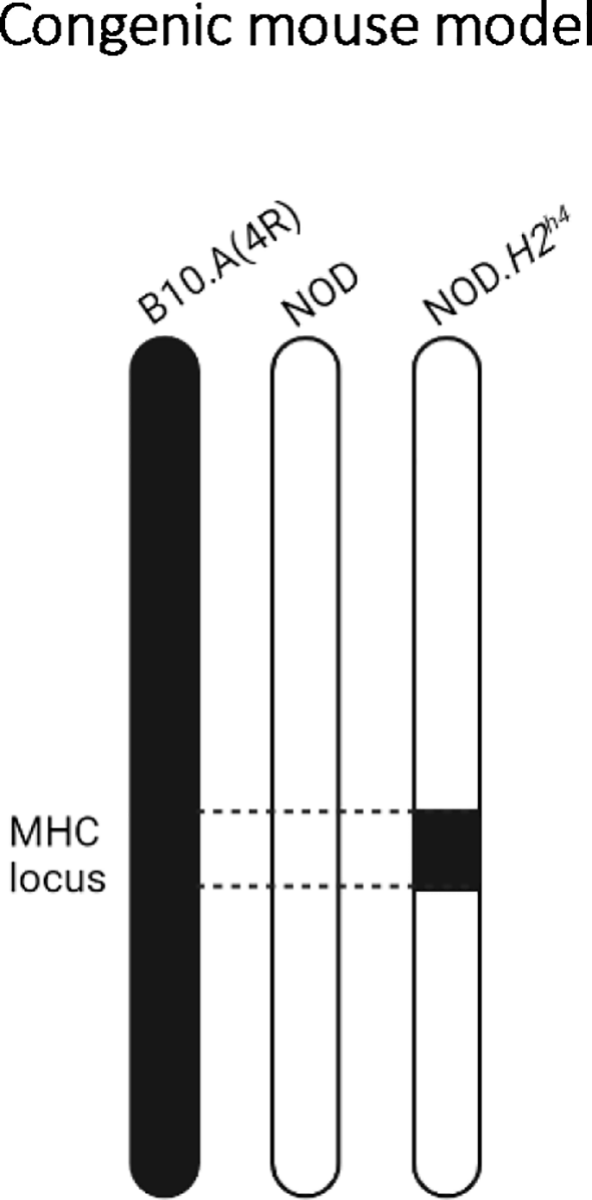
The NOD.*H2^h4^* congenic mouse model. Representation of the mouse chromosome 17 from the parental B10.A(4R) (left), parental NOD (middle), and congenic NOD.*H2^h4^* (right) strains. By backcrossing NOD mice to B10.A(4R), the thyroiditis-prone H2^h4^ MHC locus from chromosome 17 of the parental B10.A(4R) mouse has been selected at each backcross generation to replace in the NOD-derived H2^g7^ MHC locus, resulting in the NOD.*H2^h4^* congenic mouse.

NOD.*H2^h4^* congenic mice were originally generated to determine the impact of the MHC class II locus on diabetes and insulitis development (26, 27). Specifically, the NOD.*H2^h4^* bears the thyroiditis-prone H2^h4^ MHC locus from the B10.A(4R) mouse strain, composed of H-2K^k^ and H-2D^b^ for MHC class I, and I-A^k^ for MHC class II (26) (**Figure 1**). In contrast to the low ~5% to ~15% incidence of SAT in NOD mice, 50%-70% of NOD.*H2^h4^* congenic mice develop SAT (21, 26, 28). Moreover, subjecting the mice to an iodine-rich diet enhances the severity and the incidence of thyroid lesions in both NOD and NOD*.H2^h4^* congenic mice, which can reach an incidence of nearly 100% in both strains (21, 22, 24, 28–30).

While NOD.*H2^h4^* mice develop SAT, this strain is completely protected from diabetes onset (21, 26, 28). This suggests that the progression to diabetes is not necessary for SAT development, and that the break of tolerance towards thyroid autoantigens is favored by the H2^h4^ MHC haplotype, while the H2^g7^ MHC haplotype is necessary for diabetes onset. This observation also revealed that the organ-specific autoimmune susceptibility determined by the NOD genetic background can be shifted to other organs by modification of different genetic loci. In other words, different MHC loci in NOD mice can predispose to different organ-specific autoimmune diseases. Studies in families with T1D and AITD also revealed a strong genetic link to the MHC class II locus (17, 31, 32). Specifically, the MHC class II haplotype DR3-DQB1*0201 is a risk haplotype shared by both T1D and AITD (17, 31), while HLA-DR3 is specifically linked to T1D susceptibility (31, 32). Therefore, in both mice and humans, the MHC locus shifts the autoimmune response towards given target organs.

To assess the contribution of the MHC locus in thyroiditis development in mice, a comparative study was done using the NOD.*H2^h4^* mouse and the NOD.*H2^k^* mouse (28). The primary difference between the NOD.*H2^h4^* and NOD.*H2^k^* mice is the presence of I-E MHC class II molecule in the H2^k^ locus (26, 28). After exposure to an iodine-rich diet, the extent of the autoimmune thyroiditis and the levels of thyroglobulin and TPO autoantibodies were higher in the NOD.*H2^h4^* mice than in the NOD.*H2^k^* mice, in which TPO antibodies were essentially absent (28). This suggests that variants in the MHC locus between these two mouse strains influence the thyroid autoantibody profile (28). Consequently, the NOD.*H2^h4^* mouse, which develops both thyroglobulin and TPO autoantibodies, is arguably the most representative mouse model of human AITD pathology (28).

### Immune Cells Infiltration Within the Thyroid Gland

One of the key characteristics shared between AITD in humans and SAT in the NOD.*H2^h4^* mouse is the infiltration of immune cells within the thyroid gland. The recruitment and migration of lymphocytes in this gland is supported by adhesion molecules expressed on endothelial cells (33). Of interest, whereas NOD mice express high levels of ICAM-1 on thyrocytes, CBA/J, A/J, BALB/c, and C57 mice show little to no expression of ICAM-1 (33). The high expression of ICAM-1 on thyrocytes driven by the NOD genetic background (and thus also present in NOD.*H2^h4^* mice), is a genetic risk factor to SAT. ICAM-1 promotes the recruitment of immune cells into the thyroid, which then target specific thyroid autoantigens (33).

In the NOD.*H2^h4^* mouse model, as in people living with AITD, the thyroid immune cell infiltrate is predominantly composed of CD4^+^ and CD8^+^ T cells, B cells, macrophages, natural killer cells, and dendritic cells (34). Still, in humans, information regarding the kinetics of the infiltration within the thyroid is limited. To better understand the kinetics of thyroid cell infiltration, Bonita et al. took advantage of the NOD.*H2^h4^* mouse model (34). They show that the immune cell infiltration in the NOD.*H2^h4^* thyroid begins with CD4^+^ T cells, followed by CD8^+^ T cells and macrophages, and finally by B cells (34).

#### CD4^+^ and CD8^+^ T Cells

T cells are part of the adaptive arm of the immune response and self-reactive T cells are necessary and sufficient for onset and progression of many autoimmune diseases (35–38). Elimination of CD4^+^ and CD8^+^ T cells completely prevents thyroiditis development by suppressing thyroid infiltration and thyroid autoantibody production in the NOD mouse, even on iodine-supplemented diet (24). This suggests that T cells are necessary for SAT (24). In addition to promoting thyroid autoantibody production by B cells (30, 34), CD4^+^ T cells are also required for the maintenance of inflammation in the thyroid gland (30). IFN-γ, secreted by CD4^+^ T cells, damages thyrocytes (30, 34) and induces the expression of MHC class II and adhesion molecules on thyrocytes, ultimately resulting in the recruitment of other immune cells, such as CD8^+^ T cells, macrophages, B cells, and plasma cells (30, 34). CD8^+^ T cells also contribute to disease progression by secreting cytokines, namely IFN-γ and TNFα (34), and by mediating perforin/granzyme-dependent lysis of thyrocytes, resulting in severe damage to the thyroid gland (34).

#### iNKT Cells

**Figure 2:**
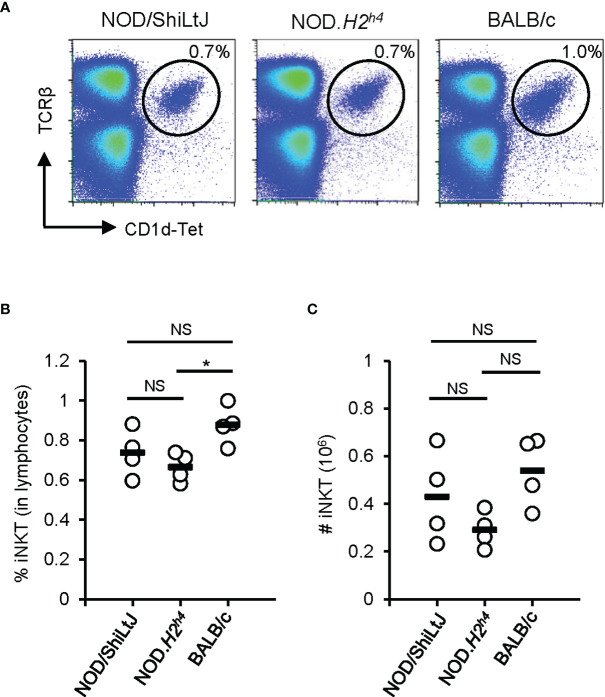
iNKT cell abundance in the spleen. Spleen of NOD/ShiLtJ, NOD.*H2^h4^*, and BALB/c mice were stained with antibodies to TCRβ and with CD1d tetramer (NIH Tetramer Core Facility). Data was acquired on BD FACSCanto II flow cytometer and analyzed with FlowJo. **(A)** Representative flow cytometry profiles of iNKT (TCRβ^+^CD1d-Tet^+^) cells in the spleen of NOD/ShiLtJ, NOD.*H2^h4^*, and BALB/c mice. **(B)** Compilation of the percentage of iNKT cells in the spleen of NOD/ShiLtJ, NOD.*H2^h4^*, and BALB/c mice (n = 4). **(C)** Compilation of absolute number of iNKT cells in the spleen of NOD/ShiLtJ, NOD.*H2^h4^*, and BALB/c mice (n = 4). One-way ANOVA tests were performed for statistical analysis. Non-significant, NS; *P-*value > 0.05, and *; *P*-value = 0.03.

Invariant Natural Killer T (iNKT) cells have first been identified as an unusual T cell population expressing both T cell receptors (TCR) and the NK markers (NK1.1, NKG2D, and Ly49) (39–41). iNKT cells recognize antigens by the non-polymorphic MHC class I-like molecule CD1d (39–41). These cells exhibit a wide array of immunological functions such as the production of chemokines and cytokines, cytolytic activity, and activation and recruitment of other cell types (39, 41). Of interest, an indirect pathogenic role of iNKT cells has been suggested in autoimmune thyroiditis (42). Sharma et al. generated two iNKT cell lines derived from NOD.*H2^h4^* splenocytes (42). After stimulation with thyroglobulin, these iNKT cell lines produce cytokines such as IFN-γ, TNF-α, IL-2, IL-4, and IL-10 (42). The adoptive transfer of thyroglobulin-stimulated iNKT cell lines enhanced autoimmune thyroiditis in NOD.*H2^h4^* mice fed with an iodine-rich diet (42), suggesting that iNKT cells enhanced autoimmune thyroiditis in NOD.*H2^h4^* mice. In addition, it was reported that the spleen of NOD.*H2^h4^* mice contains more iNKT cells than BALB/c mice (43), suggesting a link between iNKT cell abundance and SAT susceptibility. With the availability of CD1d-tetramers allowing to quantify iNKT cells more precisely, we revisited this concept. In contrast to the previous report (43), we observed a higher percentage and number of iNKT cells in the spleen of BALB/c mice when compared to NOD.*H2^h4^* mice (**Figure 2**). Further studies are required to understand the true implication of these cells in the development of autoimmune thyroiditis.

#### Regulatory T Cells (Treg) and T-Helper (Th) Cells

Tregs are immunomodulatory cells that prevent autoimmune responses and thus could be used as a therapeutic in autoimmune diseases (44). Accordingly, a depletion of CD25^+^ Tregs before subjecting the mice to an iodine-rich diet increases the severity of thyroiditis in NOD.*H2^h4^* mice (45), suggesting an important role for Tregs in the control of autoimmune thyroiditis.

Apart from Tregs, other Th subsets differentiated from naïve CD4^+^ T cells include Th1, Th2 and Th17, which are primarily distinguished based on the expression of specific transcription factors and their cytokine profile (46). Th1, Th2 and Th17 respectively express T-BET, GATA-3 and RORγt and secrete IFN-γ, IL-4 and IL-17 as their prototypical cytokine (47). In NOD.*H2^h4^* mice, the presence of IFN-γ in the thyroid before the onset of lesions suggests that Th1 cytokines may play an important role in the initiation of autoimmune thyroiditis (30, 34). In addition, Th2 cytokines, such as IL-4 and IL-13, are maximal after thyroid lesions develop suggesting that these cytokines are involved in the late chronic phase of the disease, maintaining the thyroid inflammatory response (30). Moreover, NOD.*H2^h4^-*IFN-γ^-/-^, NOD.*H2^h4^-*IFN-γR^-/-^, and NOD.*H2^h4^-*IL-17^-/-^ are resistant to the development of thyroiditis (48, 49), suggesting that both Th1 and Th17 profiles contribute to the pathology (50).

Of interest, there is an interplay between Tregs and Th cells in immune responses (51). This holds true in susceptibility to thyroiditis. Indeed, while both NOD.*H2^h4^*.IL-17^-/-^ and NOD.*H2^h4^* IFN-γR^-/-^ mice are resistant to thyroiditis, depletion of CD25^+^ Tregs induces thyroiditis in NOD.*H2^h4^*.IL-17^-/-^ mice but not in NOD.*H2^h4^* IFN-γR^-/-^ mice (50). This suggests that Tregs may more effectively control Th1-driven thyroiditis than Th17-driven pathology. Altogether, these observations point to a key role for Th cells in the development and progression of thyroiditis. Knowing that Th subsets facilitate the humoral response (46), they may effectively contribute to autoantibody production in thyroiditis.

#### B Cells

**Figure 3:**
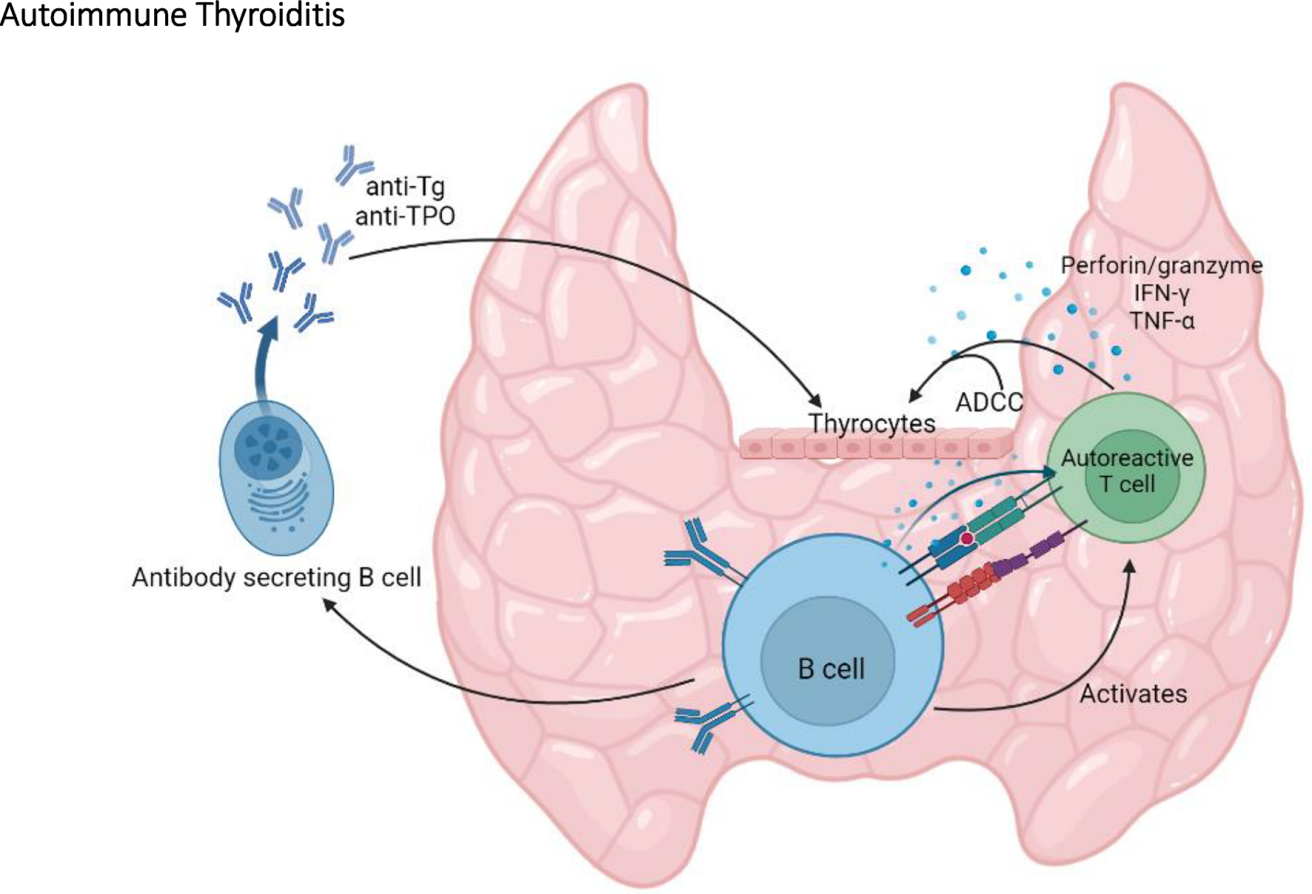
The central role of B cells in autoimmune thyroiditis. By producing anti-thyroglobulin (Tg) and anti-TPO autoantibodies, by activating autoreactive T cells, and by promoting antibody dependent cell cytotoxicity (ADCC), B cells play an important role in the onset and the progression of autoimmune thyroiditis in the NOD mouse.

By producing antibodies, B cells can provide immune protection against infections (52). However, B cells can also have pathogenic roles in autoimmune diseases by producing autoantibodies, by promoting immune complexes deposition, antibody dependent cell cytotoxicity (ADCC), and as antigen-presenting cells (APCs) (53). Indeed, B cells are important players in SAT in the NOD.*H2^h4^* mouse. This is exemplified in the NOD.*H2^h4^-μ^-/-^* mouse, devoid of B cells, as well as in NOD.*H2^h4^* mice treated with anti-IgM or anti-CD20 antibodies, to deplete B cells (54–56). In these models, B cell depletion results in a decrease in the severity of thyroid lesions, as well as undetectable levels of thyroid autoantibodies (54–56). Further characterization of B cells in NOD.*H2^h4^* mice revealed that expression of costimulatory molecules, such as CD80 and CD86, is increased on B cells following SAT onset (56). In addition, these B cells produce proinflammatory cytokines such as TNF-α and IL-6 (56). By providing costimulatory signals and secreting proinflammatory cytokines, it was suggested that B cells act as APCs, promoting the activation and expansion of autoreactive T cells (54–56). This model proposes a central role for B cells in autoimmune thyroiditis *via* their involvement in the activation of pathogenic T cells and their production of autoantibodies (**Figure 3**). Incidentally, B cells are essential for the development of Graves’ disease in which hyperthyroidism is directly caused by thyroid stimulating antibodies that target the TSHR (13).

### Production of Thyroid Autoantibodies

In addition to immune infiltration, the breakdown of tolerance towards thyroid autoantigens is shared between autoimmune thyroiditis in humans and NOD.*H2^h4^* mice, as shown by the presence of autoantibodies. In both species, the major thyroid autoantigens are thyroglobulin (13), the predominant component of the thyroid gland, and TPO; both thyroglobulin and TPO are involved in the process of thyroid hormones synthesis (57). The break of tolerance towards these two thyroid autoantigens can be explained by their immunogenicity (13). For example, the abundance and size of the thyroglobulin and TPO proteins promote the generation of a large pool of peptides, which can be presented on MHC to T cells (57). In mice, thyroglobulin autoantibodies appear first followed by TPO autoantibodies (28, 29), suggesting that thyroglobulin is one of the first targeted autoantigens (57). The NOD.*H2^h4^* mouse, when exposed to iodine-supplemented diet, develops thyroglobulin-antibodies of subclasses IgG1 and IgG2b (30). IgG2b thyroglobulin antibodies correlate with thyroid lesions and could therefore represent a biomarker for predicting thyroiditis (29). Of interest, treating NOD.*H2^h4^* mice with blocking antibodies to PD-1 and to CTLA-4 markedly enhances thyroiditis and autoantibodies to thyroglobulin and TPO (58).

In humans, antibody levels to thyroglobulin and TPO are twice as high in women than in men, with reported values of 15.2 U/ml in women vs 7.6 U/ml in men for thyroglobulin and 17 U/ml in women compared to 8.7 U/ml in men for TPO (14). However, in NOD.*H2^h4^* mice, the levels of thyroglobulin antibody levels are higher in males than females (22), whereas TPO antibody levels are higher in females than males (22). Thus, the presence of TPO antibodies in NOD.*H2^h4^* mice more closely resembles the situation in humans than the presence of thyroglobulin antibodies (22). Of note, autoantibodies to thyroglobulin or to TPO in NOD.*H2^h4^* mice are species specific, and do not cross-react with human thyroglobulin or human TPO (30, 57). Importantly, most humans with autoantibodies to thyroglobulin and TPO are euthyroid. Hypothyroidism is only manifest after extensive thyroid lymphocytic infiltration and thyroid tissue damage depletes the substantial thyroid hormone reserves and overwhelms the capacity of TSH to restore thyroid function (14, 59). Consequently, like NOD.*H2^h4^* mice, most patients with autoantibodies to thyroglobulin and/or TPO have subclinical disease (14). It should be emphasized that autoantibodies to thyroglobulin and particularly to TPO are markers of thyroid lymphocytic infiltration and are a risk factor for the development of hypothyroidism (60, 61). Of note, as for autoimmune diabetes, the presence of autoantibodies directed towards thyroid antigens reflects an ongoing humoral response. Yet, the direct pathogenic potential of these autoantibodies has not been clearly demonstrated, except for thyroid stimulating antibodies that target the TSHR in Graves’ disease (13). To that effect, transgenic expression of the human TSHR A-subunit at low levels in the thymus enables hTSHR/NOD.*H2^h4^* females, exposed to iodine-supplemented diet, to develop stimulating antibodies to the TSHR, the hallmark of Graves’ disease (62). These TSHR antibodies stimulate cAMP production by human-TSHR-expressing cells in a bioassay. However hTSHR/NOD.*H2^h4^* mice do not develop hyperthyroidism, because the antibodies target human TSHR and do not cross react with the mouse TSHR (63).

Overall, the NOD.*H2^h4^* mouse has presented itself as an invaluable mouse model for the study of AITD and manifestations of this disease, such as immune cell infiltration and autoantibody production; these traits are similar to those observed in people living with AITD. The NOD.*H2^h4^* mouse strain therefore represents an excellent animal model for the dissection of the mechanisms leading to AITD (30) and for the investigation of potential therapies against autoimmune thyroiditis (21, 22, 28, 45, 64). Moreover, manipulation of this mouse model has revealed that thyroiditis results from complex immune responses, where T cells are necessary for disease progression. Still, the humoral arm of the immune response plays a clear role in this pathology, as the presence of autoantibodies precedes disease diagnosis and eliminating B cells dampens the pathology. There is also evidence to support a role for B cells in antigen presentation to T cells. All of these traits are reminiscent of autoimmune diabetes progression in NOD mice, suggesting a parallel between the organ-specific immune mechanisms leading to these two pathologies.

## Neuropathies

In the general population, the prevalence of neuropathy, also called peripheral neuropathy, is around 2% and increases with age up to 8% in people older than 55 years old (65). Peripheral neuropathy is characterized by damage to the axon or myelin of a neuron (66). In contrast, polyneuropathy (PNP) describes a pathology where several nerves of the peripheral nervous system are damaged, such as sensory, motor, and/or autonomic nerves (66). PNPs, with a prevalence of ~5% to 8% (67), are the most common type of peripheral nervous system disorder and are caused by various factors, such as chronic alcoholism, chemotherapeutic drugs, genetic factors, and vitamin deficiency or overdose (66, 67). In Europe and North America, diabetes remains the most common cause of PNP, with diabetic patients representing from 30% to 66% of all PNP cases (65–67). Notably, more people are affected by diabetic neuropathy (DN) than all other types of PNP, including Charcot-Marie-Tooth, Guillain-Barré syndrome, and chronic inflammatory demyelinating polyneuropathy (65–67). Indeed, DN affects from 200 to 600 individuals per 100 000 people each year, whereas the prevalence is less than 15 in 100 000 individuals for all other PNPs combined (66).

DN is a painful disease defined by loss of sensory function and sensation of numbness, prickling, or burning in the distal lower extremities (66, 68). In people living with diabetes, the exact cause of these neuropathic symptoms is unknown, but some hypotheses involve metabolic, neurovascular or autoimmune pathways (69–71). The more common hypothesis suggests that chronic elevation of glucose level in the blood of people living with diabetes leads to redox imbalance and ultimately to oxidative stress (71, 72). This oxidative stress leads to glycation and oxidation of proteins, as well as dyslipidemia characterized by low levels of high-density lipoprotein cholesterol and high levels of total cholesterol, triglycerides, and low-density lipoprotein cholesterol. Dyslipidemia reduces blood flow and nerve perfusion, possibly resulting in neuropathic symptoms (71).

It is estimated that around 50% of people living with diabetes will develop DN (68). Concomitant with the increase in diabetes prevalence, the prevalence of DN is also increasing (73) but remains similar between PWT1D (11–50%) and people living with type 2 diabetes (PWT2D) (8–51%) (68). The incidence of DN is higher in PWT2D (6,100 per 100,000 person-years) than in PWT1D (2,800 per 100,000 person-years) (68). This difference between prevalence and incidence occurring in PWT1D and PWT2D could be due to several factors like differences in the age of diabetes development (68).

### The NOD Mouse as a Model of Autoimmune Neuropathy

As for PWT1D, NOD mice are also prone to develop autoimmune damage to the nervous system. Indeed, autoimmune reactions occurring in NOD mice can shift from the pancreatic islets towards nervous tissues after inhibition or disruption of costimulatory pathways, cytokines, or transcription factors that are important in the maintenance of immune tolerance. Here we will discuss some genetically modified NOD mice that develop autoimmune neuropathy and therefore represent a tool for the study of this disease.

### Disruption of Immune Tolerance Leading to Autoimmune Neuropathy

**Figure 4:**
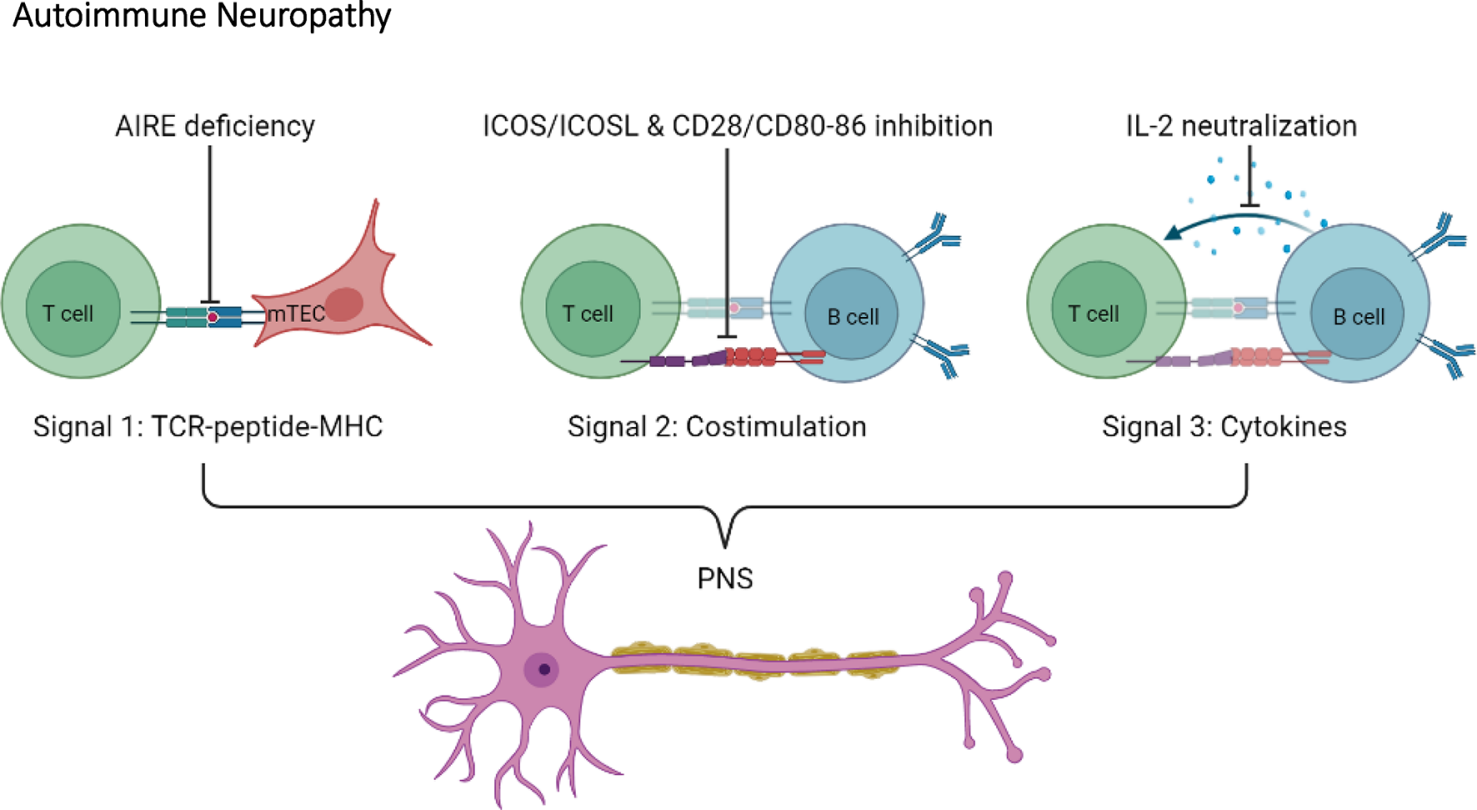
Disruption of T cell activation signals induce autoimmune neuropathy in the NOD mouse. Autoimmune neuropathy can be induced in the NOD mouse after modulation of one of the three T cell activation signals. More specifically, mutations in AIRE transcription factor expression in medullary thymic epithelial cells (mTECs) indirectly impact signal 1. In addition, disruptions in co-stimulation signals, such as ICOS/ICOSL and CD28/CD80-86 (signal 2), or neutralization of IL-2 (signal 3) induce autoimmune neuropathy in the NOD mouse.

T cell activation requires three different signals: signal 1; TCR signaling *via* recognition of peptides presented by MHC, signal 2; costimulatory molecules, and signal 3; cytokines (74, 75). As mentioned, T cells are necessary for autoimmune diabetes progression in NOD mice. In trying to understand how T cells contribute to autoimmune diabetes, various NOD mouse models where genetically engineered to specifically target signal 1, 2 or 3. Altering either signal 1, 2 or 3 in NOD mice appears to shift the pancreatic β cell-specific autoimmune response towards the nervous system (**Figure 4**).

#### AIRE Transcription Factor: An Indirect Impact on Signal 1

The transcription factor AIRE promotes the ectopic expression of tissue-restricted antigens in the thymus (76, 77). The presentation of these self-antigens allows for the negative selection of self-reactive thymocytes and favors the generation of Tregs that mediate peripheral tolerance (78–80). The NOD.Aire^GW/+^ mouse has a dominant G228W mutation in the gene coding for AIRE causing a partial loss of function, such that expression levels of tissue restricted antigens is reduced by 10% relative to NOD mice (81, 82). Of interest, the NOD.Aire^GW/+^ mouse shows a decrease in the thymic expression of myelin protein 0, one of the major autoantigens of the peripheral nervous system, representing more than 50% of the peripheral myelin protein content (83, 84). In addition to revealing that myelin protein 0 expression in the thymus is regulated by AIRE, it also explains the loss of tolerance to this protein in the NOD.Aire^GW/+^ mouse (82, 83). Indeed, the partial loss of AIRE function in NOD.Aire^GW/+^ mice promotes the escape of myelin protein 0 self-reactive T cells into the periphery, which target nervous system elements but also pancreatic tissue (82). This results in the development of autoimmune peripheral neuropathy, similar to human chronic inflammatory demyelinating polyneuropathy, as well as autoimmune diabetes (82, 83). Therefore, a slight shift in the abundance of self-antigen expression in the thymus of NOD.Aire^GW/+^ mice indirectly impacts signal 1, by not providing sufficient self-antigen presentation to the developing thymocytes. This, in turn, allows for the escape of self-reactive T cells, some of which target the nervous system, causing peripheral neuropathy.

#### Costimulatory Pathways: Signal 2

Costimulatory molecules are expressed at the surface of immune cells and enhance the intracellular signal provided by signal 1. CD28 is the prototypical costimulatory molecule for naïve T cell stimulation (85, 86). It is constitutively expressed on T cells and binds to the CD80 and CD86 receptors expressed on APCs (87). ICOS, a member of the CD28 family, is expressed on activated T cells and binds ICOSL on APCs (88). The interaction of ICOS with ICOSL and/or of CD28 with CD80 and CD86 triggers a co-stimulatory signaling cascade, which facilitates T cell activation (87). Of relevance, these costimulatory pathways are involved in autoimmunity (88, 89).

To define the involvement of ICOS and CD28 costimulatory pathways in T1D, genetic deletion of ICOS, ICOSL or CD86 was performed in NOD mice. Interestingly, NOD.ICOS^-/-^, NOD.ICOSL^-/-^, and NOD.CD86^-/-^ mice are all protected from diabetes, suggesting an important role for these costimulatory pathways in autoimmune diabetes (90, 91). However, autoimmune neuropathies spontaneously developed in all of these strains (90, 91). Specifically, the NOD.ICOS^-/-^ and NOD.ICOSL^-/-^ mice show neuromuscular autoimmunity characterized by hind leg paralysis and immune infiltration of T cells, macrophages and granulocytes in the peripheral and central nervous system (PNS, CNS), including peripheral nerves, sensory ganglia, muscles, brain, and spinal cord (91). The reason for the autoimmunity deviation from the pancreas to nervous tissue in the NOD.ICOS^-/-^ and NOD.ICOSL^-/-^ mice remains unknown (91). Analogously, the NOD.CD86^-/-^ mouse develops a spontaneous autoimmune peripheral polyneuropathy (SAPP) (90). And, as for NOD.Aire^GW/+^ mice, NOD.CD86^-/-^ mice display a break of tolerance towards myelin protein 0, the dominant autoantigen in the peripheral nervous system (83, 84). The reasons for the shift in target organ for the autoimmune response may be explained, in part, by the fact that CD86 genetic deletion leads to overexpression of CD80 on myeloid dendritic cells infiltrating the peripheral nerves (90, 92). The overexpression of CD80 on these APCs leads to activation of myelin-specific T cells, myelin sheet destruction and SAPP development (90). In addition, disruption of the CD28 costimulatory pathway leads to a reduction in Treg number, which could ultimately enhance susceptibility to SAPP (93). Exploiting genetically modified NOD mice will help dissect how disruptions in signal 2 facilitate a shift in the autoimmune response towards a different target organ. This is especially important when considering therapeutic approaches that target these pathways, to avoid treatment of T1D that would instead lead to the development of neuropathy.

Although autoimmune diabetes and neuropathy are characterized by different manifestations, the genetic factors promoting these two diseases on the NOD genetic background partially overlap. The H2^g7^ MHC haplotype of NOD mice not only plays an important role in autoimmune diabetes but is also necessary for the development of autoimmune neuropathy in the NOD.CD86^-/-^ mouse (94). This suggests that the H2^g7^ haplotype promotes self-reactivity against various organs. The genetic susceptibility overlap can also be attributed to non-MHC loci. For example, diabetes resistance loci were introduced in NOD.CD86^-/-^ mice to generate NOD.CD86^-/–^ *Idd3/5* and NOD.CD86^-/–^ *Idd3/10/18* congenic mice. These congenic mice are completely protected from both autoimmune diabetes and neuropathy (94). Thus, genetically modified NOD mice allow to study mechanisms as well as genetic factors promoting the development of autoimmune neuropathy.

#### Cytokines: Signal 3

T cell activation is modulated by the presence of cytokines, which represent the third signal for T cells activation (75). Unbalanced cytokine production is deleterious and may lead to the development of autoimmunity (95–97). A key cytokine in modulating T cell function is IL-2; it facilitates the proliferation of T cells and is involved in immune tolerance by allowing the homeostatic maintenance of Tregs (98–100). Similar to NOD mice with targeted disruption of costimulatory molecules, autoimmune peripheral neuropathy has been described in NOD mice deficient in IL-2 (98). While intraperitoneal injection of anti-IL-2 monoclonal antibodies in NOD mice accelerates diabetes onset, it also induces the development of autoimmune peripheral neuropathy in more than 50% of the treated mice (98). This neuropathy is characterized by ataxia and paralysis of the limbs due to demyelination of the peripheral nerves (98). Anti-IL-2 treatment in NOD mice enhances autoimmunity by reducing Treg number, their activation, and their suppressive function (98). Of interest, IL-2 is one of the key candidate genes in the *Idd3* susceptibility locus (101, 102). *Idd3*, and thus IL-2 variants, may generally predispose NOD mice to autoimmune diseases by altering the function and development of Treg cells (98).

Altogether, genetic manipulations leading to alterations in T cell signal 1, 2 or 3 in NOD mice can shift the immune response from pancreatic β cells towards the nervous system. This break in T cell tolerance allows for infiltration of autoreactive T cells in the peripheral nerves, which ultimately leads to the production of autoantibodies targeting myelin protein 0 by self-reactive B cells (84). Of note, autoantibodies targeting myelin protein 0 have also been found in serum from individuals diagnosed with Guillain-Barré syndrome and chronic inflammatory demyelinating polyneuropathy (103, 104).

### Production of Autoantibodies Targeting Nervous System Antigens

Pancreatic islets are surrounded by cells of the autonomous nervous system (105). In addition, pancreatic β-cells and neuronal cells share some autoantigens such as GAD, ICA515, and the neuronal type III intermediate filament protein, peripherin (105, 106). These autoantigens of the pancreatic nervous system are targeted by islet-infiltrating autoreactive T cells as well as autoantibodies (105). The production of autoantibodies against pancreatic nervous system antigens occurs in the early phase of diabetes and could explain certain neurological pathologies occurring in the prediabetic stage in humans and mice (105). In addition, B cell producing peripherin autoantibodies have been isolated directly from the pancreatic islets of NOD mice (106). Altogether, these observations point to a potential cross-reactive autoimmune response to both pancreatic β cells and neuronal cells, resulting in the production of autoantibodies as a reflection of an ongoing humoral immune response, which likely contributes to the pathology.

To specifically study the impact of peripherin-specific B cells in diabetes and neuritis, a BCR-transgenic mouse model (NOD-PerIg) was generated (106). In this mouse, B cells express the H and L chain Ig transgene from the peripherin-specific hybridoma clone H280, isolated from the pancreas of NOD mice (107). Compared to non-transgenic NOD mice, NOD-PerIg mice develop early onset diabetes, with an expansion of diabetogenic T cells, revealing an important association between the pancreas and the nervous system (107). Genetic manipulation of B cell responses in the NOD mouse has identified a clear link between autoimmune diabetes and neuropathy. This link between autoimmune diabetes and neuropathy has also been observed in non-NOD mouse models of autoimmune diabetes (108).

In sum, as for thyroiditis, manipulating the NOD mouse has informed us on cellular processes and genetic pathways linking autoimmune diabetes to peripheral neuropathies. As multiple immune characteristics are shared between autoimmune neuropathy in NOD mice and humans, the genetically modified NOD mice described above continue to be useful to improve our knowledge on autoimmune neuropathy, as well as the connection between the pancreas and the nervous system.

## Autoimmune Biliary Diseases

Primary biliary cirrhosis (PBC), primary sclerosing cholangitis (PSC), and IgG4-associated cholangitis (IAC) represent the three main forms of autoimmune biliary diseases (ABD) (109, 110). All ABD share specific symptoms such as bile duct obliteration and cholestasis, characterized by a strong reduction of bile flow (111–114). Here we will focus on the most common form of ABD which is PBC, with an overall prevalence of ~19 to ~40 cases per 100 000 individuals depending on the geographic location (110). PBC is a chronic autoimmune cholestatic liver disease most frequently observed in middle-aged women (115), and is characterized by lymphocytic infiltration of the liver portal tracts, destruction of the epithelial cells of the intrahepatic bile duct, and serologic hallmarks of antimitochondrial autoantibodies (AMA) (116). Notably, 90-95% of people living with PBC (PWPBC) will develop AMA; these autoantibodies long precede clinical symptoms of PBC, often for many years, and yet represent one of the three criteria for the definitive diagnosis of PBC (117).

### The NOD Mouse as a Model of ABD

In an attempt to understand the contribution of genetic loci linked to autoimmune diabetes susceptibility in NOD mice, the congenic NOD.c3c4 mouse carrying resistance alleles on chromosomes 3 (*Idd3*, *Idd10*, *Idd17*, *Idd18*) and 4 (*Idd9.1*, *Idd9.2*, *Idd9.3*), was generated (118, 119). The NOD.c3c4 mouse does not show signs of autoimmune diabetes (119), but about half of the female and a quarter of the male mice spontaneously develop a fatal form of ABD (118). Similar to human PBC, NOD.c3c4 mice exhibit lymphocyte infiltration in the liver, production of autoantibodies, biliary obstruction, and finally liver failure leading to death (118–120). Of interest, the NOD.c3c4 strain was the first mouse model of human PBC (119).

#### T Cell Infiltration in the Liver

In NOD.c3c4 mice, abundant T cell infiltration can be observed in the liver, with CD4^+^ and CD8^+^ T cells primarily located in the biliary epithelium (119). CD4^+^ T cells in the liver produce pro-inflammatory cytokines such as IFN-γ and IL-2 (119). Importantly, antibody-mediated depletion of T cells leads to a significant reduction in disease onset in NOD.c3c4 mice (119). Moreover, transfer of CD4^+^ T cells from a NOD.c3c4 mouse to a lymphopenic NOD.c3c4-*scid* mouse is sufficient to induce ABD development (119). Altogether, these observations demonstrate that T cells are necessary and sufficient for ABD in NOD.c3.c4 mice.

The role of T cells in ABD development has also been investigated in a new congenic mouse model of PBC, the NOD.ABD mouse, derived from the NOD.c3c4 mouse (120). This congenic subline, with shorter resistance loci on chromosomes 3 and 4 than those in NOD.c3c4 mouse, develops ABD as well as autoimmune diabetes (120). This suggests that these two autoimmune diseases are not mutually exclusive in the NOD.ABD congenic mouse model. Of interest, the development of both T1D and PBC has also been reported in humans (121). The NOD.ABD mouse model develops a similar form of ABD as the NOD.c3c4 mouse characterized by common bile duct (CBD) dilation, immune cell infiltration, and biliary epithelial proliferation resulting in cyst formation (120). Of note, NOD.ABD mice show an accumulation of central and effector memory CD8^+^ T cells in the liver, which effectively produce IFN-γ and TNF-α (120). Additionally, the transfer of NOD.ABD CD8^+^ T cells alone or with CD4^+^CD25^-^ T cells into NOD.c3c4-*scid* mice promotes ABD development in these recipients, suggesting an important role of autoreactive CD8^+^ T cells in ABD (120). Overall, studies in NOD.ABD and NOD.c3c4 congenic mice highlight an important pathogenic role of T cells in ABD development.

#### Production of Autoantibodies in ABD

As mentioned above, the presence of autoantibodies, particularly of AMA, is a strong serologic hallmark of disease, with 90-95% of PWPBC presenting with these autoantibodies (117, 122). In NOD.ABD mice, AMA were shown to bind the E2 subunit of the pyruvate dehydrogenase complex (PDC-E2), part of the mitochondrial 2-oxoacid dehydrogenase complexes (120). While PDC-E2 is a ubiquitous autoantigen expressed in all nucleated cells in the body, in PWPBC, only bile duct epithelial cells are targeted (119). The reason for the specific targeting of bile duct epithelial cells is unclear; it suggests that other components are at play, and that the presence of AMA may be secondary to tissue destruction. Of interest, anti-PDC-E2 antibodies are present in both NOD.c3c4 and NOD.ABD mouse models of PBC (119, 120). However, the proportion of NOD.ABD mice presenting with these autoantibodies is rather low, and, in contrast to PWPBC, anti-PDC-E2 antibody-positive mice increases with disease severity and age (120). Still, anti-PDC-E2 antibodies appear before detectable liver immune cells infiltration in both PWPBC and NOD congenic mice (119, 122).

In addition to anti-PDC-E2 antibodies, antinuclear antibodies (ANAs) and anti-Smith antibodies (anti-Sm) are also observed in the sera from PWPBC but at a lower incidence (48% of PWPBC develop ANAs vs 24% for anti-Sm) (123). Notably, the presence or absence of ANAs and anti-Sm varies among the different NOD mice congenic for chromosomes 3 and/or 4. In contrast to NOD and NOD.ABD mice which do not develop ANAs and anti-Sm autoantibodies, these autoantibodies are found in the serum of NOD.c3c4 and other congenic lines (118, 120). Further investigation of congenic sublines suggests that the *Idd9.3* locus is sufficient for ANAs and anti-Sm autoantibody production (120, 124). Within the *Idd9.3* locus, there is a candidate gene encoding for CD137 (4-1BB), an inducible costimulatory molecule on T cells (124, 125). A three amino acid difference in CD137 between NOD and B10 mice results in a lower CD137 costimulatory signal in NOD mice (124, 126). This may explain why NOD mice carrying non-NOD alleles at this locus show an increased production of autoantibodies, *via* the enhanced CD137-mediated costimulation between T cells and B cells (124, 126).

**Figure 5:**
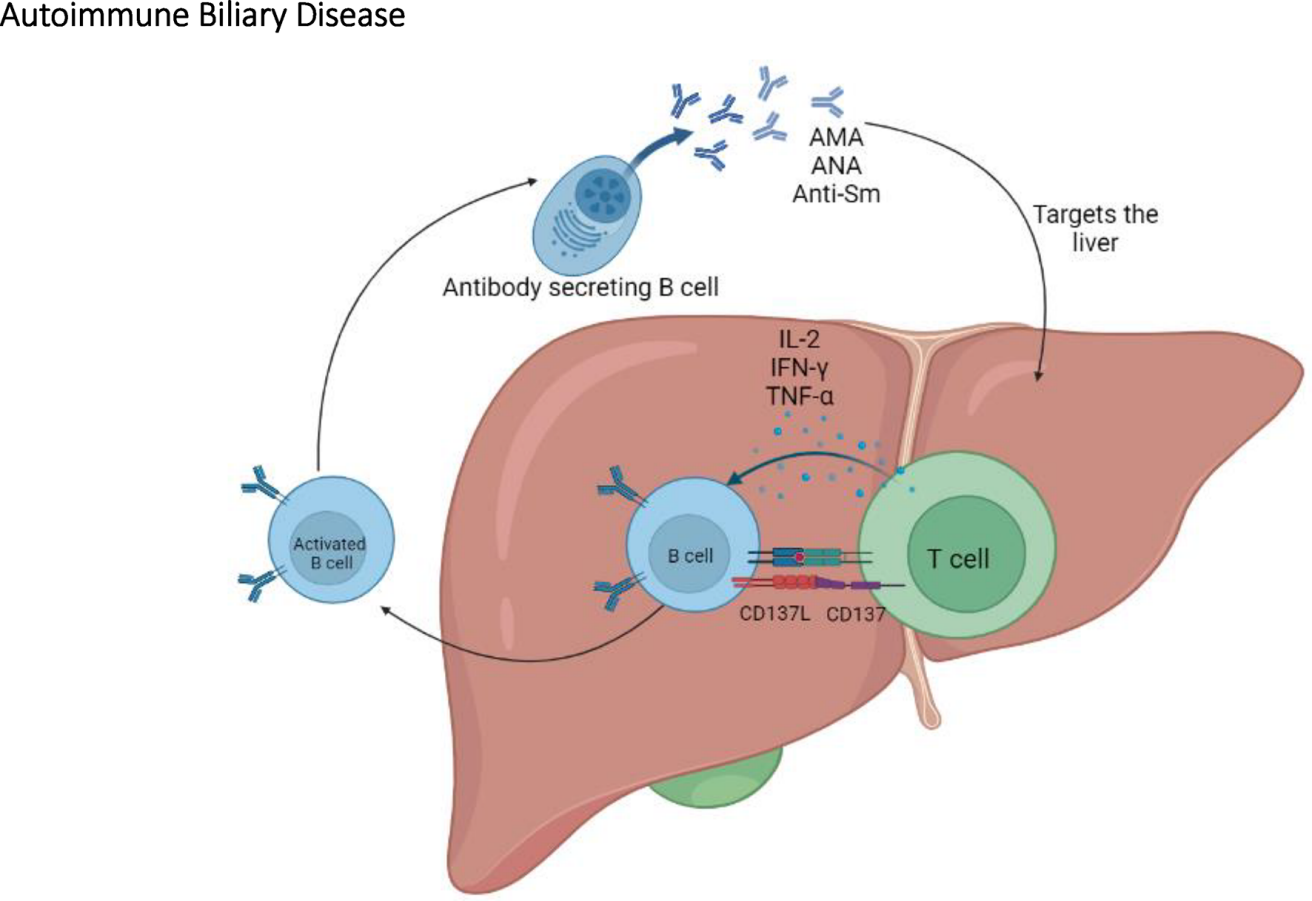
The immunopathogenesis of autoimmune biliary disease. In the NOD congenic mice, T cells play an important role in ABD by activating B cells which will eventually produce AMA, ANA, and anti-Sm autoantibodies.

Overall, the NOD.c3c4 congenic mouse is a relevant model of PBC; it shares significant characteristics with PBC including key aspects of the humoral autoantibody response (127, 128) (**Figure 5**). In addition, NOD.ABD congenic subline allows to investigate the relationship between ABD and autoimmune diabetes. These NOD congenic mice further allow the identification of relevant and possibly clinically targetable molecular pathways for the development of new treatments.

## Polyautoimmunity in NOD Mice

The term polyautoimmunity is used to describe the presence of more than one autoimmune disease in the same individual (129–131). For instance, a given NOD mouse can simultaneously present with multiple autoimmune diseases, such as autoimmune diabetes and thyroiditis (20, 132). The polyautoimmunity does not need to include autoimmune diabetes. In fact, NOD.*CCR7* ^-/-^ mice are protected from diabetes, but develop multiple autoimmune phenotypes, including immune infiltration in the thyroid, sciatic nerve, lung, stomach, intestine, uterus, and testis, among others (133). Notably, the thyroid pathology in these mice most closely resembles the primary hypothyroidism observed in humans (133). In addition, autoimmune diabetes-resistant NOD.*H2^h4^* and NOD.*H2^h4^*-IFN-γ^-/-^CD28^-/-^ mice spontaneously develop thyroiditis and Sjögren’s syndrome (SS) (134–136). While these findings further highlight the remarkable autoimmune-prone background of the NOD mouse, we will mostly focus our discussion to polyautoimmune phenotypes that include autoimmune diabetes.

In addition to autoimmune diabetes and thyroiditis (20, 132), NOD mice can present with both autoimmune diabetes and SS (137–143). T1D and SS can also co-occur in humans, with up to 55% of PWT1D exhibit symptoms of SS, such as keratoconjunctivitis sicca (dry eyes) and xerostomia (dry mouth) (144). SS is a chronic autoimmune exocrinopathy disorder characterized by lymphocyte infiltration and progressive damage to the exocrine glands, mainly the lacrimal and salivary glands (145, 146). These damages lead to decreased tears and saliva secretion, which ultimately result in keratoconjunctivitis sicca and xerostomia (145, 146). SS is notably defined by important B cell alterations of the humoral immunity which result in a polyclonal B cell activation and antibodies production (135, 147). In fact, one of the main hallmarks of SS is the presence of lymphocyte infiltration in the exocrine glands which formed organized lymphoid structures called ectopic follicles (148). In these ectopic follicles, all subsets of B cells are present, including antibody-secreting B cells which produce pathogenic antibodies that are useful for SS diagnosis (135, 148). These autoantibodies, which target non-organ-specific antigens, are Rheumatoid factor, anti-double stranded DNA, ANA, anti-Ro, and anti-La (147–149). Of note, the presence of anti-Ro and anti-La is a criterion for SS diagnosis (149). A study in NOD.*H2^h4^* mice reveals that anti-Ro and anti-La appear before the development of ectopic follicles in the salivary gland whereas antibodies to double stranded DNA only develop after the appearance of ectopic follicles (148). These observations are consistent with anti-Ro and anti-La being the hallmark of SS and particularly as markers identifying patients in the active stage of the disease. SS is most prevalent in women aged between 30 to 60 years old, with a female to male ratio from 20:1 to 9:1 (137, 146, 150). In NOD mice, as in humans, the development of SS seems to be influenced by sex hormones because SS in NOD mice is significantly higher in females than males (137).

Another example of polyautoimmune traits present in humans is T1D and multiple sclerosis (MS) (151). MS is an autoimmune inflammatory disease of the central nervous system characterized by autoimmune responses against the protective myelin sheaths around nerve fibers, leading to severe and progressive neurological impairment (152). PWT1D have a 3 to 20 times higher risk of developing MS compared to the general population (151, 153–156). In addition, these two autoimmune diseases share some genetic and environmental susceptibility factors (151). Exposure to vitamin D seems to protect against the onset of MS and T1D (157). Interestingly, immune responses against pancreatic islet have been observed in people with MS, and, conversely, PWT1D show immune responses against central nervous system antigens (158). In addition to T1D and MS polyautoimmunity, T1D can also be observed in association with other autoimmune diseases such as AITD and DN in the same individual (15–18, 68).

**Figure 6:**
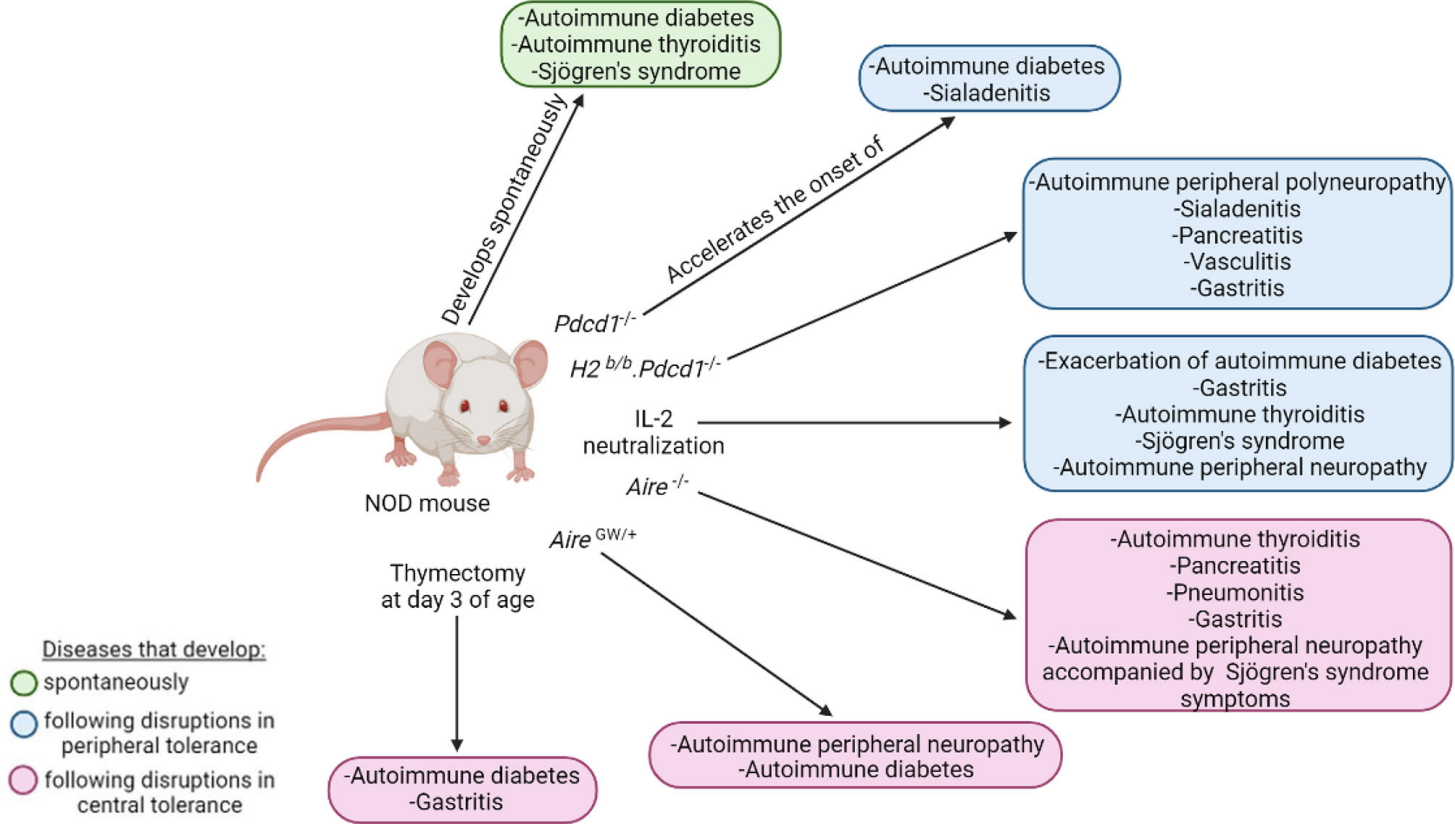
Polyautoimmunity in the NOD mouse. The NOD mouse can spontaneously develop autoimmune diabetes, autoimmune thyroiditis, and Sjögren’s syndrome (green). Disruptions in peripheral (blue) and central tolerance (pink) exacerbate or induce a wide spectrum of other organ-specific autoimmune diseases.

The polyautoimmunity observed in NOD mice allows investigation of the mechanisms underlying this complex trait. Indeed, polyautoimmunity in NOD mice can be exacerbated by genetic manipulation and/or modulation of immune functions (**Figure 6**). For instance, targeting PD-1, AIRE, IL-2 or performing thymectomy in NOD mice promotes polyautoimmunity, as discussed below.

### PD-1 Driving Polyautoimmunity

PD-1, which is coded by the *Pdcd1* gene, is an immunoreceptor involved in the regulation of peripheral tolerance by inducing and maintaining T cell clonal anergy and homeostatic control of B cells and myeloid cells (159–163). The interaction of PD-1 with its ligands, PD-L1 and PD-L2, suppresses immune responses like autoimmunity and sustained inflammation (163, 164). As such, PD-1 deficiency on the NOD genetic background accelerates the onset and incidence of autoimmune diabetes, with an onset at 5 weeks instead of 12-17 weeks in NOD mice, and an incidence reaching 100% by 10 weeks (164). Sialadenitis is also accelerated and more severe, with significantly greater pathological scores at 6 weeks of age in NOD.*Pdcd1^-/-^* relative to NOD mice (164). Sialadenitis is an inflammation of the salivary glands caused by an increase in the activation and effector functions of autoreactive T cells (165). The NOD.*Pdcd1^-/-^* mouse, which presents a rapid onset of both autoimmune diabetes and sialadenitis, can thus be used to study polyautoimmunity. The early onset of autoimmune diabetes in the NOD.*Pdcd1^-/-^* mice is in part due to early and severe insulitis, resulting in rapid destruction of pancreatic β-cells (164). As for NOD mice, insulitis and autoimmune diabetes in NOD.*Pdcd1^-/-^* mice is dependent on the H2^g7^ MHC locus. Indeed, as for NOD-*H2^b/b^* mice (26), NOD-*H2^b/b^.Pdcd1^-/-^* mice are completely protected from insulitis and autoimmune diabetes (160, 166). This indicates that the H2^g7^ haplotype is absolutely required for autoimmune diabetes development, even in NOD.*Pdcd1^-/-^* mice (26, 160). Rather than developing autoimmune diabetes, the NOD-*H2^b/b^* mice develop SS (141, 167–169), whereas the NOD-*H2^b/b^.Pdcd1^-/-^* female mice are polyautoimmune; they develop spontaneous peripheral polyneuropathy, sialadenitis, pancreatitis, vasculitis, and gastritis (160, 166). This polyautoimmunity is likely due to a break in T cell tolerance as a consequence of a disruption of the PD-1 pathway (160). To identify the genetic factors that drive this polyautoimmune phenotype, Jiang et al. performed a genetic linkage analysis between NOD-*H2^b/b^.Pdcd1^-/-^* and C57BL/6.*Pdcd1^-/-^* mice (166). They identified 14 non-MHC quantitative trait loci linked to these autoimmune traits (166). These studies highlight the relevance of using genetically manipulated NOD mice to study polyautoimmunity to identify additional genetic variants linked to autoimmune diseases (160, 166).

### AIRE Transcription Factor as a Prototypical Factor Causing Polyautoimmunity

As mentioned above, the AIRE transcription factor has an important role in maintaining self-tolerance and preventing autoimmunity (78, 170–173). The polyautoimmune syndrome resulting from AIRE mutations is a rare autosomal recessive disease called autoimmune polyendocrinopathy-candidiasis-ectodermal dystrophy (APECED) or autoimmune polyendocrine syndrome type-1 (174–176). In addition to developing chronic mucocutaneous candidiasis, hypoparathyroidism, and primary adrenal insufficiency, people living with APECED also develop several organ-specific autoimmune manifestations including T1D, autoimmune thyroiditis, gastritis, and hepatitis (170, 177, 178). As in people living with APECED, AIRE deficiency in mice from various genetic backgrounds, including NOD.*Aire^-/-^* mice, have circulating autoantibodies targeting multiple organs and lymphocytic infiltration in various tissues, representing a good model for APECED studies (178, 179). Of note, NOD.*Aire^-/-^* mice are protected from autoimmune diabetes but exhibit thyroiditis, pancreatitis, pneumonitis, gastritis, and autoimmune peripheral neuropathy accompanied by the development of some SS symptoms (146, 167, 180). As mentioned above, the NOD.*Aire* ^GW/+^ mouse is also polyautoimmune in that it develops autoimmune peripheral neuropathy and autoimmune diabetes (82, 83). Thus, both the NOD.*Aire^-/-^* and NOD.*Aire* ^GW/+^ mice are relevant models to study polyautoimmunity.

To study the impact of the humoral response in polyautoimmunity, Gavanescu et al. compared NOD.*Aire^-/-^* and NOD.*Aire^-/-^* μMT mice, and showed that the lack of B cells in AIRE-deficient mice strongly reduces autoimmune manifestations such as organ inflammation (179). In addition, depleting B cells in a variant of the NOD.*Aire^-/-^* model significantly reduced inflammation and destruction of the pancreas (179). These results suggest that B cells contribute to APECED pathology and that anti-B cell therapies could help alleviate symptoms in people living with APECED (179).

### IL-2 and Polyautoimmunity

Polyautoimmunity is also observed in NOD mice treated with IL-2 neutralizing antibodies (98, 166). These mice show an exacerbation of autoimmune diabetes and develop a wide spectrum of organ-specific autoimmune diseases such as gastritis, thyroiditis, SS, and peripheral neuropathy (98). This is likely due to the fact that IL-2 neutralizing antibodies broadly reduce Treg number, as well as their suppressive functions (98–100, 181).

### Thymectomized NOD Mice Develop Polyautoimmunity

As for PD-1 deficiency, AIRE mutations, and IL-2 neutralization, thymectomy (Tx) performed at three days of age (d3-Tx) in NOD mice leads to the development of polyautoimmunity (182). While d3-Tx in NOD mice does not impact autoimmune diabetes onset, it concomitantly results in autoimmune gastritis development (182). Autoimmune gastritis is a CD4^+^ T cell-mediated disease mainly characterized by lymphocytic infiltration in the gastric mucosa and the production of autoantibodies against the parietal cell H+/K+ ATPase proton pump (183–185). The BALB/c mouse is particularly susceptible to autoimmune gastritis (183). D3-Tx BALB/c mice develop autoimmune gastritis that closely resembles human disease and for which the pathologic score is higher than in d3-Tx NOD mice (183). Interestingly, susceptibility loci linked to autoimmune gastritis, namely *Gasa1* and *2*, are located on mouse chromosome 4 (184, 185) and overlap with the *Idd11* and *Idd9* loci, respectively (183–185). This suggests a strong genetic association between autoimmune gastritis and diabetes (186). Notably, the prevalence of autoimmune gastritis in PWT1D is 3-to-5-fold higher than in the general population (187). The polyautoimmunity developing in Tx mice is thus relevant to autoimmune gastritis and diabetes.

Overall, disturbances in various components affecting T cell tolerance exacerbates polyautoimmunity in NOD mice, providing clues to the development of polyautoimmunity and potentially revealing therapeutic targets to alleviate the severity of the pathologies.

## The NOD Mouse as a Relevant Tool Beyond Spontaneous Autoimmunity

Unarguably, the NOD mouse model is a useful tool to study autoimmune diabetes. By genetic manipulation, derivatives of the NOD mouse model represent relevant spontaneous models for multiple human autoimmune pathologies. However, one cannot ignore other highly relevant uses of the NOD mouse model. For one, intravenous injection of pertussis toxin in NOD mice induces the development of experimental autoimmune encephalitis (158). This new induced model exhibits phases of remission, and closely mimics clinical and histopathological properties of MS; it may help to determine the genetic and environmental factors that promote the progression of MS (158). In addition, following injection of heat-killed bacillus Calmette-Guérin, NOD mice develop a non-organ specific autoimmune rheumatic disease similar to SLE (188–190), creating yet another relevant induced model to study the progression of a human pathology.

Apart from autoimmune diseases, the NOD strain has been used for studying human cells. Indeed, due to a polymorphism in CD172a, the NOD strain allows for better engraftment of human hematopoietic cells than other mice (191, 192). The strong interaction between the CD172a protein on the NOD macrophages and CD47 on human cells leads to a negative regulation of macrophage phagocytosis (193). Engraftment of human cells is typically performed in NOD.SCID, NOD.Rag^-/-^, NOD.SCID.IL2Rγ^-/-^ or NOD.Rag^-/-^.IL2Rγ^-/-^ mice, deficient in various components of the adaptive immune system, to further facilitate xenogeneic engraftment (194–199). Additional NOD mouse models are constantly being created to enhance human cell engraftment or to study specific diseases (200–206). For instance, the Human Immune System (HIS)-DRAGA (HLA-A2.HLA-DR4.Rag1^-/-^.IL-2Rγc^-/-^.NOD) mouse, grafted with human epithelial cells expressing the human angiotensin-converting enzyme 2 (hACE2) receptor in their lungs, was generated for COVID-19 research (207). Following immune reconstitution with human HLA-matched hematopoietic stem cells and intranasal infection with SARS-CoV-2, the HIS-DRAGA mouse exhibits T cell infiltration in the lungs and develops the different forms of severity of COVID-19 disease, as seen in the human population (207). The HIS-DRAGA mouse strain provides an important model for studying SARS-CoV-2 infection, as well as the immune responses generated against this virus, and can be used to test potential therapeutics and vaccines (207).

## Conclusion

The NOD mouse remains one of the best models to study T1D. It is useful to study autoimmune susceptibility as well as genetic and cellular factors contributing to breakdowns of immune tolerance. Genetic manipulation of the NOD mouse has generated excellent models for studying spontaneous organ-specific autoimmune diseases other than diabetes such as thyroiditis, neuropathies, ABD and even polyautoimmunity. The manifestation of these autoimmune diseases in the NOD variant strains share many characteristics with human diseases, particularly immune cell infiltration in the targeted organ and a strong humoral response involving the generation of autoantibodies. Although pancreatic β cells have been shown to be particularly fragile in NOD mice (208), the exact reasons why the NOD mouse develops autoimmune diabetes whereas genetically modified NOD mice spontaneously develop autoimmune responses to other target organs remain unknown. In addition to organ-specific autoimmune diseases, disturbances in peripheral or central tolerance in NOD mice lead to polyautoimmunity, providing key information on the importance of these immune tolerance mechanisms for maintaining health. All in all, the NOD mouse, along with the several NOD congenic mice and NOD genetic knockout mice that have been generated over the years, represent indispensable tools in research that may be exploited for applications much broader than the study of type 1 diabetes. With their close parallel to various human autoimmune pathologies, these models should be exploited to increase our understanding of these specific pathologies as well as to design and test novel therapeutics.

## Author Contributions

A-MA wrote the first draft of the manuscript and prepared most of the figures. FL-V contributed to the first draft of the manuscript and prepared some figures. RC analyzed the data for **Figure 2**. HA generated the data for **Figure 2** and revised the final version of the manuscript. SM supervised HA and revised the final version of the manuscript. SL supervised A-MA and FL-V and revised the final version of the manuscript. All authors contributed to the article and approved the submitted version.

## Funding

A-MA holds scholarships from the Canadian Institutes of Health Research and the Fondation de l’Hôpital Maisonneuve-Rosemont. FL-V holds scholarships from the Fondation de l’Hôpital Maisonneuve-Rosemont, the Cole Foundation, and the Fonds de Recherche Quebec Santé. Funding for studies by SMM: Supported by NIH DK 54684 (SMM) and DK 19289 (Basil Rapoport). SL is a Research Scholars Emeritus awardee from the Fonds de Recherche Quebec Santé.

## Conflict of Interest

The authors declare that the research was conducted in the absence of any commercial or financial relationships that could be construed as a potential conflict of interest.

## Publisher’s Note

All claims expressed in this article are solely those of the authors and do not necessarily represent those of their affiliated organizations, or those of the publisher, the editors and the reviewers. Any product that may be evaluated in this article, or claim that may be made by its manufacturer, is not guaranteed or endorsed by the publisher.
